# Primary central nervous system lymphoma presenting as bilateral uveitis in an immunocompetent hepatitis C virus+ patient: a case report

**DOI:** 10.1186/1757-1626-2-152

**Published:** 2009-10-13

**Authors:** Guerriero Silvana, Lorenza Ciraci, Domenico Santorsola

**Affiliations:** 1 Department of Ophthalmology and ORL, University of Bari, Piazza Giulio Cesare 11, Bari, 70124, Italy; 2 Department of Onco-hematology AUSL, BAT1, viale Padre Pio, Trani, 70059, Italy

## Abstract

**Background:**

To report a case of masquerade syndrome presenting as bilateral uveitis in an HCV positive patient, and to highlight the difficulties in distinguishing between chronic uveitis and malignancy-induced inflammation.

**Case report:**

In January 2005 a 54-year-old Caucasian man was referred to the Ophthalmological Department for bilateral visual loss, severe vitritis, and a significant cataract in both eyes. His clinical history was significant for HCV infection. The uveitis treated with low dose of steroids and immunosuppressors, yielding a partial remission of the symptoms. One year later he developed a Primary Central Nervous System Lymphoma. In January 2007 he returned to our department for cataract surgery. The patient underwent phacoemulsification of the cataract in the right eye, intraocular lens implantation and intravitreal injections of 4 mg triamcinolone acetonide. After one month fundus biomicroscopy showed a solid lesion at the posterior pole, consistent with a retinal relapse of the Primary Central Nervous System Lymphoma. Restaging investigations were unremarkable and ruled out a disease relapse, and a diagnostic vitrectomy showed only rare inflammatory cells. In view of the progressive swelling of the retinal lesions we decided to treat the patient with intravitreal Methotrexate. Complete remission of the retinal lesions with retinal scarring was achieved after 12 months. In May 2008 the patient underwent phacoemulsification of the cataract in the left eye and intraocular lens implantation. A vitreal tap was performed and was positive for rare abnormal cells CD45+, CD20-. Vitreous sampling did not yield enough cells for a diagnosis of monoclonality. No systemic or intravitreal therapy was performed because of the absence of central nervous system relapses, the small number of atypical cells found in the vitreous sample and the absence of retinal masses. After three months the patient developed a central nervous system relapse of the lymphoma and rapidly died.

**Conclusion:**

In elderly patients suffering from uveitis a masquerade syndrome should always be suspected. Vitreous sampling may not yield enough cells for diagnosis and the vitritis may be steroid-sensitive, at least initially. This makes a differential diagnosis between chronic uveitis and malignancy-induced inflammation very difficult.

## Background

The incidence of true immune-mediated uveitis declines in the elderly. Infection endophthalmitis, especially arising after surgery, and malignancy occur at higher frequency. In this age group, the suspicion of a masquerade syndrome, particularly a primary CNS non-Hodgkin's lymphoma, should always be raised [1]. We describe a case of an immunocompetent HCV+ patient referred to the Ophthalmological Department of Bari University Hospital for bilateral uveitis. The inflammation, initially diagnosed as a complication of the chronic hepatitis C virus, was, instead, the first manifestation of a primary central nervous system lymphoma, that was diagnosed one year later.

## Case presentation

In January 2005 a 54-year-old Caucasian immunocompetent man was referred to the Ophthalmological Department for bilateral visual loss (right eye: 20/100 and left eye: 20/60), severe vitritis, cells in the anterior chamber and a significant cataract in both eyes. In both eyes the intraocular pressure (IOP) was 15 mmHg.

His clinical history was significant for HCV infection treated with interferon, and for insulin dependent diabetes. The bilateral ocular uveitis was correlated to the HCV infection and treated with low dose of steroids and immunosuppressors, yielding a partial remission of the symptoms. One year later the patient developed neurological symptoms, (a slow-down in mental and motility performance and a central defect of the VII cranial nerve) that led to a cranial CT examination and to the diagnosis of Primary Central Nervous System Lymphoma (PCNSL) arising from the left nuclei of the base, the nucleus caudatus, the anterior arm of the internal capsule, the insula and thalamus(stereotaxically diagnosed as CD45+, CD20+, CD3neg, CD10neg, Td Tneg, ki-67/MIB-1 = 90%), treated with L-VAMP multi chemotherapy and radiotherapy, resulting incomplete recovery.

In January 2007 he returned to our department for a deterioration of the visual acuity(only light perception in the right eye and 20/200 in the left eye). He presented a total white cataract in the right eye and a severe cataract affecting the visus in the left eye. There were rare inflammatory cells in the anterior chamber of both eyes. Ultrasonographic examination showed a severe vitritis and a small, solid uplift at the posterior pole, misinterpreted as macula cystoids edema in the right eye and a mild vitritis in the left eye.

**Figure 1:**
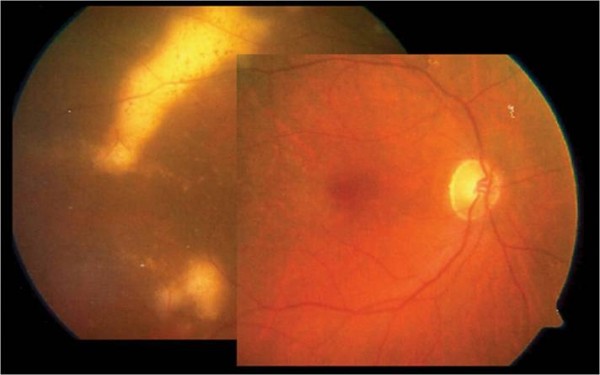
**Baseline**. Fundus biomicroscopy one month after cataract surgery and intravitral injection of triamcinolone acetonide revealed solid, irregular whitish lesions at the posterior pole, consistent with a retinal relapse of the PCNSL.

The patient underwent phacoemulsification of the cataract in the right eye, acrylic hydrophilic intraocular lens implantation and intravitreal injections of 4 mg triamcinolone acetonide. After one month, best corrected visual acuity in the right eye was 20/40, IOP was 17 mmHg, fundus biomicroscopy showed a solid, irregular whitish lesion at the posterior pole temporally to the macula, consistent with a retinal relapse of the PCNSL (Figure 1).

Restaging investigations (CT, MRI, spinal tap, osteomedullary biopsy) were unremarkable and ruled out a disease relapse, and a vitreal tap and subsequent diagnostic vitrectomy showed only rare inflammatory cells.

**Figure 2:**
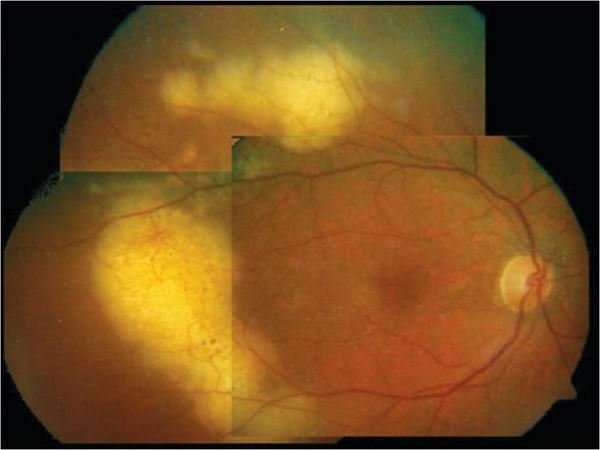
**Fundus biomicroscopy 45 days after cataract surgery showed a progressive swelling of the retinal lesions**.

In view of the progressive swelling of the retinal lesions (Figure 2), the absence of indications for systemic therapy and the risk of visual loss secondary to radiotherapy, we decided to treat the patient with local chemotherapy in the form of intravitreal Methotrexate(IMTX), as suggested by other authors [2,3].

**Figure 3:**
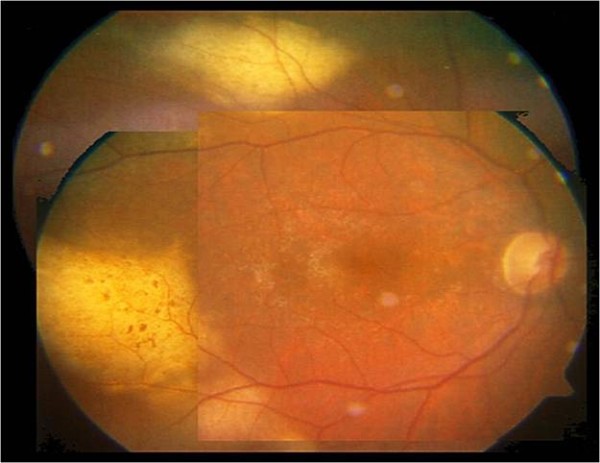
**Fundus biomicroscopy 15 days after the first IMTX revealed a partial regression of the retinal lymphomatous infiltrates**.

**Figure 4:**
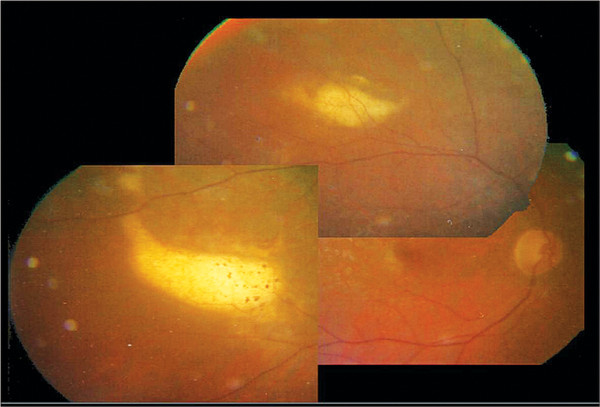
**Fundus biomicroscopy after 8 IMTX**.

**Figure 5:**
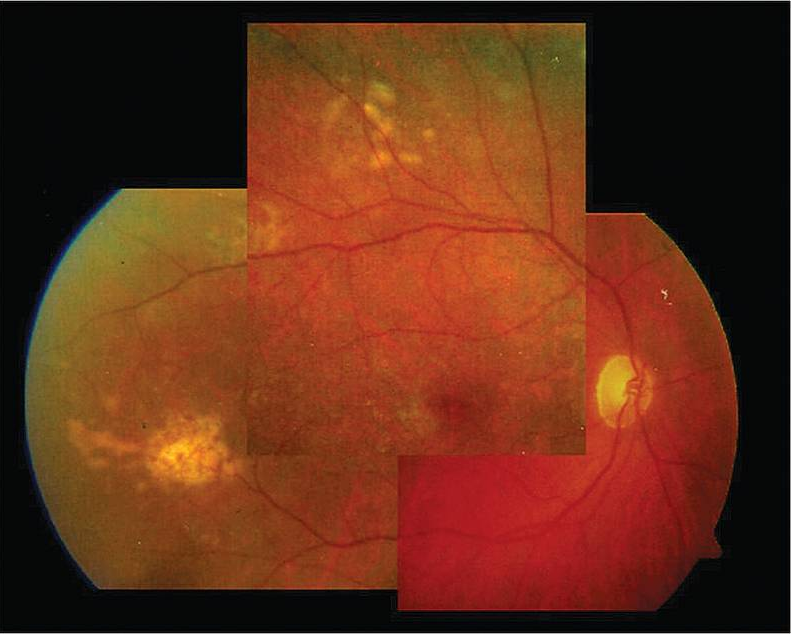
**Fundus biomicroscopy after 12 IMTX revealed a further regression of the malignancy**.

**Figure 6:**
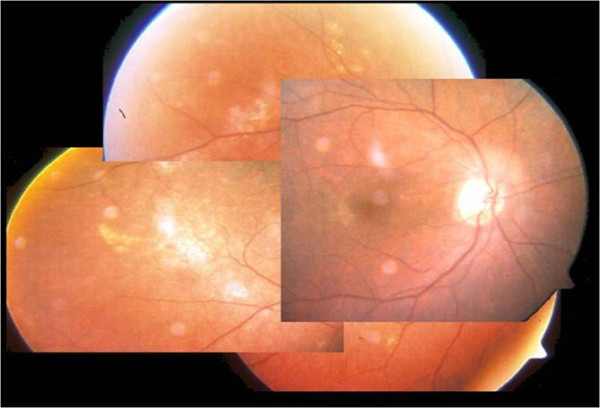
**Fundus biomicroscopy: Complete remission of the retinallesions was achieved after 12 months of IMTX therapy, when the retinal masses had beencompletely replaced by retinal scars (25 IMTX)**.

After obtaining written informed consent, surgery was performed, together with full asepsis, preoperative cleaning of the conjunctival sac with povidone-iodine 5%, topical anesthesia with topical benoxinate HCL 0.4% drops. Then 400 μg/0.1 ml of IMTX were intravitreally injected via the pars plana using a 30 gauge needle. Eight twice weekly intravitreal methotrexate injections were administered during the induction phase, followed by eight weekly consolidation injections. Subsequently, a maintenance phase involved nine monthly methotrexate injections to complete one year of treatment (25 injections). At each visit, measurement of the visual acuity (VA) and intraocular pressure (IOP) was made, as well as slit lamp biomicroscopy to view individual cells within the vitreous cavity, and dilated fundoscopy to see malignant cellular infiltrates involving the retina. Gonioscopic examination was added to the monitoring protocol to identify iris and anterior chamber angle neovascularisation. The retinal lesions showed a progressive, rapid regression as from the first administration of the intravitreal therapy (Figure 3, Figure 4 and Figure 5). After 12 months the retinal lesions had been replaced by retinal scarring (Figure 6), best corrected visual acuity in the right eye increased to 20/20. We did not observe any ophthalmic or systemic side effects related to IMTX, such as corneal epitheliopathy, maculopathy, vitreous hemorrhage, optic atrophy or sterile endophthalmitis. Restaging investigations (TC, RMN, spinal tap, osteomedullary biopsy) were performed periodically and ruled out a disease relapse.

In May 2008 the patient underwent phacoemulsification of the cataract in the left eye and acrylic hydrophilic intraocular lens implantation. A vitreal tap was performed and was positive for rare abnormal cells CD45+, CD20-. Vitreous sampling did not yield enough cells for a diagnosis of monoclonality, the hallmark of malignant processes.

After one month, visual acuity in left eye was 20/20, there was a mild vitritis and no retinal lesions. A new restaging of the disease was performed and no relapse was detected. No systemic or intravitreal therapy was performed because of the absence of central nervous system relapses, the small number of atypical cells found in the vitreous sample and the absence of retinal masses.

Unfortunately, after three months the patient developed a central nervous system relapse of the lymphoma and rapidly died of the malignancy and the toxic effect of the new high dosage systemic chemotherapy.

## Discussion

Primary central nervous system lymphoma (PCNSL)accounts for 4 to 7% of brain tumours. It occurs with a markedly increased incidence in the immunosuppressed population but its incidence has increased in the past two decades in immunocompetent patients as well [4-6], probably due to the better diagnostic imaging and biopsy sampling methods available in recent years.

Primary intraocular lymphoma (PIOL), is a subset of primary central nervous system lymphoma (PCNSL), in which lymphoma cells invade the subretinal pigment epithelial space and vitreous cavity, with or without central nervous system involvement at the time of the ocular diagnosis. The age of onset of PIOL ranges from 15 to 85 years, with a mean age of the late 50s to early 60s. Both sexes are affected but most series point to a higher incidence in women.

Typical clinical symptoms of PIOL include blurred vision and floaters. In many of these patients uveitis is initially diagnosed, especially if there is no known history of cerebral involvement. The most common ophthalmic manifestations are similar to those seen in patients with posterior uveitis or vitreitis, combined anterior and posterior uveitis, or subretinal pigment epithelial infiltrates [7].

Without treatment, PIOL is often rapidly fatal, especially when associated with PCNSL. Currently, the five year survival rate ranges from less than 20% to 34% with the best therapy [8,9]. It is imperative, then, to make a prompt diagnosis so that life extending treatment may be started. The diagnosis of intraocular lymphoma can be very difficult patients with intraocular lymphoma may seem perfectly well with no systemic or neurological symptoms and signs, peripheral blood, bone marrow cytology and cellular morphology are usually normal and therefore unhelpful. Ocular examination can be helpful, but the degree of vitritis is highly variable and retinal lesions may or may not be found. Even in the presence of retinal lesions, doing a biopsy is hazardous, may miss the lesion, and if successful, will only yield a small number of cells. A vitreous sample can also be unhelpful, as in our patient, because it will only yield a small number of atypical cells so it is not possible to determine the monoclonality, or it may yield only inflammatory cells. This makes the differentiation between chronic inflammation and intraocular lymphoma very difficult [10].

In our case, the diagnosis was delayed because initially the ocular inflammation was correlated to the hepatitis C. The patient presented only few ocular signs and seemed perfectly well, with no systemic or neurological symptoms and signs. Moreover, the ocular vitritis showed a partial response to the mild steroid and immunosuppressive therapy instituted.

Only one year later the patient developed neurological symptoms, (a slow-down in mental and motility performance and a central defect of the VII cranial nerve) that led to a cranial CT examination and to the diagnosis of central nervous system lymphoma.

When the correct diagnosis of the ocular pathology was made, after extracting the cataractin the right eye few therapeutic options were available, because the patent had just undergone systemic chemo and radiotherapy, and refused eye enucleation.

Intrathecal therapy was not recommended because of the complete absence of relapse of the CNS disease. Eye radiotherapy could cause eye dryness, actinic retinopathy, optic neuropathy and resulting irreversible severe visual loss.

The response to IMTX was fast and an improvement of the visual acuity and a regression of the retinal lesions were observed within 8 weeks. We did not observe any ophthalmic side effects such as corneal epitheliopathy, maculopathy, vitreous hemorrhage, optic atrophy or sterile endophthalmitis.

Despite the good results of the systemic chemotherapy, radiotherapy and intravitreal therapy in the right eye, and the negative results of all the precedent restaging examinations, after three months the patient developed a new, fatal PCNSL localization. This case confirms the very poor prognosis of these malignancies, especially in cases of relapse.

## Abbreviations

PCNSL: Primary Central Nervous Lymphoma; PIOL: Primary Intra Ocular Lymphoma; IMTX: intravitreal Methotrexate; IOP: intraocular pressure; VA: visual acuity.

## Consent

Written informed consent was obtained from the patient's son for the publication of this case report and accompanying images. A copy of the written consent is available for review by the Editor-in Chief of this journal.

## Competing interests

The authors declare that they have no competing interests.

## Authors' contributions

SG performed cataract surgery and intravitreal therapy, analyzed the patient ophthalmological data and was a major contributor in writing the manuscript. LC performed the photographic documentation of the retinal lesions. DS analyzed and interpreted the patient data regarding the haematological disease. All authors read and approved the final manuscript.
